# Supplementary material for: Saccharomyces boulardii (CNCM I-745) improves intestinal damage in sepsis by remodeling intestinal flora structure
Source: Front Cell Infect Microbiol. 2025 Jul 31;15:1602792. doi: 10.3389/fcimb.2025.1602792 (PMC12350329; doi:10.3389/fcimb.2025.1602792)
Supplement: Supplementary file 1 [file DataSheet1.zip › summary/summary/6_Taxonomy_community/1_Taxonomy_profiling/3_Class/Group/Class_abund_top30.Group.stacked_bar.pdf]

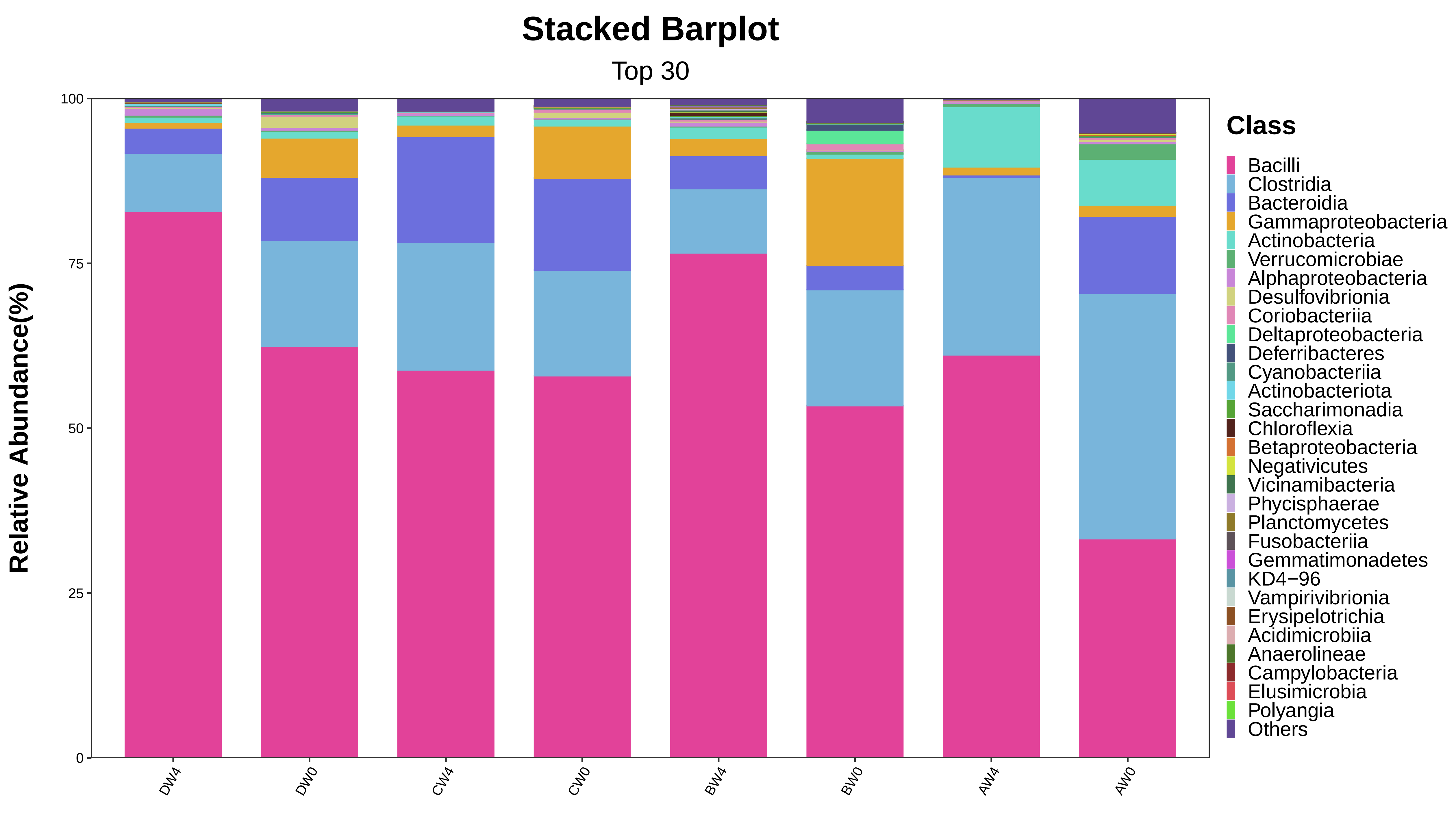
Stacked Barplot
Top 30
Relative Abundance(%)
100
75
50
25
0
DW4
DW0
CW4
CW0
BW4
BW0
AW4
AW0
Class
Bacilli
Clostridia
Bacteroidia
Gammaproteobacteria
Actinobacteria
Verrucomicrobiae
Alphaproteobacteria
Desulfovibrionia
Coriobacteriia
Deltaproteobacteria
Deferribacteres
Cyanobacteriia
Actinobacteriota
Saccharimonadia
Chloroflexia
Betaproteobacteria
Negativicutes
Vicinamibacteria
Phycisphaerae
Planctomycetes
Fusobacteriia
Gemmatimonadetes
KD4−96
Vampirivibrionia
Erysipelotrichia
Acidimicrobiia
Anaerolineae
Campylobacteria
Elusimicrobia
Polyangia
Others
